# Supplementary material for: Is the digital economy empowering high-quality tourism development? A theoretical and empirical research from China
Source: PLoS One. 2024 May 2;19(5):e0303087. doi: 10.1371/journal.pone.0303087 (PMC11065292; doi:10.1371/journal.pone.0303087)
Supplement: Attachment — Submitted filename: PLOS ONE.docx [file pone.0303087.s002.docx]

Dear Authors

It is a great work.

However, I suggest you including the names of sources more often than you did. This way we could know the scholars without the need to turn up/down pages.

The, some errors to correct:

Line 71- 73. Finally, the heterogeneity effect is fully considered, and the research sample is divided into different province sizes and different DE levels to be tested empirically respectively. In order that it can provide useful reference for the HQDT. (These two sentences should be joined)

Line 168. multiple businesses or products.By effectively using the accumulated … (insert a space between the sentences)

Line 266. It uses ~~his~~  ITS strong drive and sense to produce correlation effect

Line 295. through a big data monitoring platform to enhance external response capabilities. DI as a

significant contributor to the efficiency of China's economic growth [46]. (Is it a sentence or a subheading?)

line 361. what is this word: industrial digitizationy

line 568. Although this study selected 19 sub-divisional indicators to measure the level of HQDT in each province. However,… (join these sentences)
