# Supplementary material for: Is the digital economy empowering high-quality tourism development? A theoretical and empirical research from China
Source: PLoS One. 2024 May 2;19(5):e0303087. doi: 10.1371/journal.pone.0303087 (PMC11065292; doi:10.1371/journal.pone.0303087)
Supplement: Attachment — Submitted filename: Response to Reviewers.docx [file pone.0303087.s004.docx]

**Reply to the comments**

Dear Editor and Reviewers,

Thanks for your letter concerning our manuscript entitled "Is the digital economy empowering high-quality tourism development? A theoretical and empirical research from China". We thank you for the time and effort that you have put into reviewing the previous version of the manuscript. Those comments are all valuable and helpful for revising and improving our paper. We have studied all comments carefully and have made conscientious corrections. Revised portions are marked in the paper. We would like also to thank you for allowing us to resubmit a revised copy of the manuscript. The responses to the comments are as follows:

**Reviewer 1**

The authors revised and corrected their article. Almost perfect.

**Comment :** I recommend some articles to cite. More than 70 citations are needed.

https://journals.sagepub.com/doi/10.1177/14673584231198414 and

https://www.emerald.com/insight/content/doi/10.1108/TRC-11-2022-0028/full/pdf and

https://gtg.webhost.uoradea.ro/PDF/GTG-3-2021/gtg.37309-711.pdf

Etc.

**Response:** Sincerely, thanks for your suggestion. Based on your suggestions, we have added your recommended articles in the revised version, as follows:

39. Roziqin Ali, Kurniawan Alferdo Satya, Hijri Yana Syafriyana, et al. Research trends of digital tourism: A bibliometric analysis[J]. Tourism Critiques: Practice and Theory, 2023, 4(1-2): 28-47, https://doi.org/10.1108/TRC-11-2022-0028.

45. Ives Gutierriz, João J Ferreira, Paula O Fernandes. Digital transformation and the new combinations in tourism: A systematic literature review[J]. Tourism and Hospitality Research, 2023(0), https://doi.org/10.1177/14673584231198414.

47. Setiawan Priatmoko, Lóránt Dénes Dávid. Winning tourism digitalization opportunity in the indonesia CBT business[J]. GeoJournal of Tourism and Geosites, 2021, 37(3): 800-806, https://doi.org/10.30892/gtg.37309-711.

**Reviewer 3**

Dear authors

I am afraid, there was some misunderstanding.

**Comment :** I asked the authors not to have digits (at least not as many) when referring to a source. E.g., If tourism enterprises are considered as the supply side, the AI in the growth phase is influencing their employment, costs, and management practices [21]. Do the respected authors think that we are going to turn pages up and down to see that it was Wang yueying (by the way, why is the surname/name in lower case?). Instead of this frustrating writing (NOT only here but in the entire text) the manuscript should have said: As Wang yueying states/mentions/claims/says/notices/emphasizes [21], if tourism enterprises are considered as the supply side, the AI in the growth phase is influencing their employment, costs, and management practices. Lines 89 to 110 demands 13 times of up/down of pages. I will be surprised to see a person doing that, especially the author who you are citing in the manuscript without paying enough respect to his/her achievements.

**Response:** We sincerely appreciate the valuable comments. We apologize for our misunderstanding. Based on your detailed comments and to fulfill the formatting requirements of PLOS ONE, we borrowed the citation format of Li and Yang (Li Hao, Yang Zihan. Does digital economy development affect urban environment quality: Evidence from 285 cities in China[J]. Plos One, 2024, 19(2): e0297503, https://doi.org/10.1371/journal.pone.0297503). When we cited the authors' achievements, we added the author's source to each citation. It is as follows:

“At the theoretical level: Microscopically, digital intelligence technology can establish and maintain a good relationship between consumers and tourism enterprises by providing high quality services to further improve consumer satisfaction (Wang, 2022) [20]. If tourism enterprises are considered as the supply side, the AI in the growth phase is influencing their employment, costs, and management practices (Rodolfo and Chris, 2010) [21]. Dimitra et al. argue that big data can improve the efficiency, productivity, and profitability of tourism enterprises and can provide differentiated, rich, and convenient experiences to consumers (Dimitra et al., 2020) [22]. ” Etc.

In addition, we sincerely thank you for the valuable comments. We have also provided data, verified ORCID iD, checked references, and uploaded images to PACE as required by PLOS ONE.

Thanks again to the editor and the reviewers for the time and effort on this paper. These comments are excellent and help us a lot to improve our articles! We sincerely hope that the revised manuscript will be accepted for publication in PLOS ONE.
